# The effects of typical ageing on cognitive control: recent advances and future directions

**DOI:** 10.3389/fnagi.2023.1231410

**Published:** 2023-07-28

**Authors:** Melissa Dexter, Ori Ossmy

**Affiliations:** Centre for Brain and Cognitive Development, Department of Psychological Sciences, Birkbeck, University of London, London, United Kingdom

**Keywords:** cognitive control, executive functions, ageing, planning, cognitive decline, cognitive development

## Abstract

Cognitive control is one of the most fundamental aspects of human life. Its ageing is an important contemporary research area due to the needs of the growing ageing population, such as prolonged independence and quality of life. Traditional ageing research argued for a global decline in cognitive control with age, typically characterised by slowing processing speed and driven by changes in the frontal cortex. However, recent advances questioned this perspective by demonstrating high heterogeneity in the ageing data, domain-specific declines, activity changes in resting state networks, and increased functional connectivity. Moreover, improvements in neuroimaging techniques have enabled researchers to develop compensatory models of neural reorganisation that helps negate the effects of neural losses and promote cognitive control. In this article on typical ageing, we review recent behavioural and neural findings related to the decline in cognitive control among older adults. We begin by reviewing traditional perspectives and continue with how recent work challenged those perspectives. In the discussion section, we propose key areas of focus for future research in the field.

## 1. Introduction

One of the most fundamental human skills is cognitive control—the ability to regulate and coordinate one’s thoughts, actions, and emotions to achieve goals and adapt to changing environmental demands. Lifespan research has focussed on the emergence of cognitive control from infancy (Zelazo et al., 2004) and in recent years, it has become a pressing research goal to identify what behavioural, neural, and computational factors drive developmental improvements in cognitive control (Lindenberger, 2014).

Cognitive control has been defined as an inherently variable dual-mechanism framework that allows individuals to switch between proactive and reactive control strategies, or modes, to adapt to the needs of a task or goal (Braver and West, 2008; Braver, 2012; Grady, 2012). Others have highlighted the role of cognitive control in the regulation and allocation of cognitive resources to process information (Fan, 2014). Because of the vast amount of information transmitted both internally (physiologically) and from the external environment, cognitive control is necessary for continuously adapting responses according to present goals (Braver, 2012; Fan, 2014). Environmental manipulations have been found to cause major changes in cognitive-control strategies and corresponding brain regions, and models like these are important for considering age-related changes in cognitive strategy (Braver, 2012). Even more so when cognitive demands are high during times of increased competition for resources, conflict, or distraction (Ludwig et al., 2010; Korotkevich et al., 2015).

However, understanding how cognitive control fades is as essential as understanding its emergence. The decline in cognitive control with ageing affects foundational skills in daily life, such as preserving independence, completing job-related tasks, and maintaining a healthy active lifestyle (Wiles et al., 2012; Bacsu et al., 2014; Denburg and Hedgcock, 2015; Strout et al., 2018). As a society we must promote quality of life alongside longer living, therefore it is critical to ensure sufficient knowledge to protect and conserve cognitive control in older adults, and to support the efforts of researchers and healthcare professionals to develop interventions and treatments (Lindenberger, 2014).

During the past two decades, a growing body of literature in psychology and neuroscience has challenged traditional perspectives on the decline of cognitive control with age, emphasising the heterogeneity in research data and the role of brain plasticity during ageing (Lövdén et al., 2010a; Bherer et al., 2013; Park and McDonough, 2013), as well as shifts in cognitive control strategies to compensate for age-related neurocognitive changes (Jimura and Braver, 2010). We begin this article by reviewing traditional perspectives and continue with how recent advances affect those perspectives. Finally, we propose key areas of focus for future research in the field. Atypical ageing is beyond the scope of this short review.

## 2. Traditional perspectives on ageing of cognitive control

Cognitive control has been used interchangeably with executive processes, executive functioning, or executive control (Jurado and Rosselli, 2007; Vaughan and Giovanello, 2010; Diamond, 2013). The exact sub-domains under this umbrella term remain under debate (Jurado and Rosselli, 2007; Maldonado et al., 2020), but some of the core behavioural processes have been identified as inhibitory control (inhibiting or stopping pre-potent responses or resistance to interference), mental task or set shifting (the ability to quickly shift between two goals), memory updating (relating to holding and updating relevant information in working memory), and coordination (for example, aiding motor control; Verhaeghen, 2011; Zelinski et al., 2011; Maldonado et al., 2020). These processes are at the core of ageing research because they dominate vital aspects of life quality, including mental and physical health, career, academic ambitions, social cohesion, and the fostering of relationships (Jurado and Rosselli, 2007; Diamond, 2013).

### 2.1. The global decline approach

Past research has argued for a global decline of cognitive control, typically underlined by slowing processing speed with age (Sorel and Pennequin, 2008). This deceleration in information processing affects cognitive skills, such as attention, memory, and executive functions (Korotkevich et al., 2015), making it harder to keep up in tasks that require simultaneous processing of multiple pieces of information (Li et al., 2001; Verhaeghen and Cerella, 2002). The cause of this decline relates to changes in brain structure and function over time (Adnan et al., 2019), and factors such as decreased blood flow and oxidative stress (Glade, 2010; Insel et al., 2012). Due to slow processing, older adults are also less likely to encode new information effectively, causing difficulties in retrieving information from long-term memory (Salthouse et al., 2003; Salthouse, 2009; Spreng and Turner, 2019).

Global declines also affect working memory—the ability to temporarily hold and manipulate information in mind. Working memory declines with age (Borella et al., 2010; Köstering et al., 2016), impacting a variety of daily activities, such as decision making and problem solving (Royall et al., 2005; Vaughan and Giovanello, 2010). The decrease in working memory limits the amount of information that can be actively processed, making it harder to switch between tasks and manipulate information to solve problems (Thornton and Dumke, 2005; Spieler et al., 2006).

As individuals age, their cognitive resources become increasingly taxed, leading to greater attention costs, particularly in tasks requiring cognitive resources such as attention shifting. Some researchers have proposed that performance level in two tasks simultaneously is a predictor of cognitive impairments in older adults (Downey et al., 2022). While researchers have pointed to divided attention—the ability to attend to multiple tasks simultaneously—as the source of this decline (McDowd and Craik, 1988; Craik et al., 1996; Verhaeghen and Cerella, 2002), selective attention—the ability to ignore unrelated information when performing a task—is vulnerable to ageing due to differences suppressing or filtering task-irrelevant information (Gazzaley et al., 2005; Schmitz et al., 2010; Jost et al., 2011). Additionally, difficulties in cognitively demanding situations have been related to an overall slowing of the cognitive-control network (Sorel and Pennequin, 2008; Wunsch et al., 2017) as shown in cases where older adults manage attentional control poorly during situations with salient distraction (Mayas et al., 2012).

Finally, older adults exhibit a decline in their ability to ignore irrelevant information or inhibit impulses to focus on relevant information (Hasher and Zacks, 1988; Reuter-Lorenz et al., 2021). Age has also been associated with retaining information that is no longer task relevant, which can hinder or benefit performance depending on the task (Amer et al., 2016, 2022). Inefficient inhibitory control leads to more distractibility and decreased attentional control. For example, older adults perform poorly when required to suppress prepotent actions of dominant responses (Park and Reuter-Lorenz, 2009; Zelinski et al., 2011). A large-scale cross-sectional study featuring more than 3,000 older adults suggested that the effects of ageing on inhibitory control result from a decline in fine motor skills, processing speed, and visuospatial abilities (Hoogendam et al., 2014) which are strong early markers for the general decline in cognitive control (Wunsch et al., 2017; Glover et al., 2021).

### 2.2. The frontal lobe hypothesis

At the neural level, evidence points to the pre-frontal cortex (PFC) as the central brain region that is responsible for cognitive control (Vaughan and Giovanello, 2010; Park and McDonough, 2013). The PFC is involved in a wide range of functions, including working memory (Diamond, 2013; Köstering et al., 2016), decision making (Craik and Bialystok, 2006; Denburg and Hedgcock, 2015), attentional regulation (Diamond, 2013), regulation of thoughts (Vaughan and Giovanello, 2010), and the processing of conflicting information and resolution of cognitive conflicts (Mayas et al., 2012).

Evidence linking PFC activation and cognitive control led to the frontal lobe hypothesis of ageing (West, 1996; Reuter-Lorenz et al., 2021). The main argument in this hypothesis is that the frontal lobe has a greater decline with age compared to other brain regions and it deteriorates at an accelerated rate (Rosselli and Torres, 2019), thereby leading to poorer cognitive control in older adults. Supporting empirical evidence includes structural and functional changes in the frontal lobe as people age (Park and McDonough, 2013; Cabeza et al., 2018), specifically decreased neural activity in the PFC (Jimura and Braver, 2010) and decreased grey matter volume (Rosselli and Torres, 2019). Because the PFC is one of the first areas to show decreased activity when under strain (e.g., during high stress, depression, or lack of sleep), it has been identified as a “warning system” for cognitive ageing (Diamond, 2013).

The frontal lobe hypothesis is also supported by ageing changes in neurotransmitter systems which are involved in the regulation of PFC functions. For example, the dopamine system is known to play a role in executive functioning, working memory, and attention (Zelazo et al., 2004; Park and Reuter-Lorenz, 2009), and ageing is associated with decreased dopamine modulation in the PFC, which contributes to age-related declines in cognitive control (Grady, 2012; Gutchess, 2014; Lindenberger, 2014). Other important neurotransmitters involved in PFC maturation and its effects on cognitive control are the acetylcholine (involved in attention, learning, and memory; Blokland, 1995) and norepinephrine systems (involved in attention, arousal, and the regulation of mood).

## 3. Recent advances: challenging traditional perspectives

### 3.1. Heterogeneity of the ageing cognitive-control system

For more than a decade, researchers have challenged the idea of a global cognitive decline by emphasising the heterogeneity found in the ageing research data (Lindenberger and Baltes, 1997; Wilson et al., 2002; Raz et al., 2005; Verhaeghen, 2011). While attention allocation and inhibitory control may decline with age (Rozas et al., 2008; Zanto and Gazzaley, 2017), working memory and reasoning abilities may be preserved or even improved (Hoogendam et al., 2014; Maldonado et al., 2020). Older adults even triumph over younger adults in certain crystallised cognitive control tasks, specifically remembering task-relevant information in a real-world multiple errands task (Kliegel et al., 2007). High variability was also found in the onset of decline across different cognitive-control processes (Jurado and Rosselli, 2007), and the onset of declines could be exaggerated by cross-sectional data due to cohort effects, such as age-related similarities in education level (Nyberg et al., 2012).

Heterogeneity can depend on the experimental tasks. For example, a recent meta-analysis indicated that common tasks used to measure cognitive control, such as Stroop and flanker, do not show reliable age-related deficits, whereas go/no-go and stop-signal tasks are associated with impairments in inhibitory control (Fan, 2014; Rey-Mermet and Gade, 2018). Even within inhibitory control, impairments depend on the type of task used (Borella et al., 2009; Mayas et al., 2012). These cross-task differences suggest that the ageing process of cognitive control is not a univariate reduction in performance but rather a more complex, unsynchronised change among cognitive processes.

Significant heterogeneity is also observed across individuals due to variability in socio-economic status (SES) and quality of living factors such as education, lifestyle, and general health (Migeot et al., 2022). Processes that are sensitive to stress or environmental adversity (e.g., attention allocation, emotional regulation, and decision making) are affected more by differences in SES compared to other processes. These effects relate to the limited access of individuals to quality education, healthcare, and other protective resources, alongside increased exposure to environmental toxins and other life stressors that can have a negative impact on brain function. Historically, protective factors, such as higher education and occupation attainment levels, have been discussed as promoting individual cognitive reserve levels (Stern, 2002). Reuter-Lorenz and Park (2014) suggested that factors such as education level, physical fitness, and multilingualism could protect cognitive functioning by improving brain structure and function and providing neural and cognitive support against age-related cognitive declines. Likewise, individuals with higher levels of education (Lenehan et al., 2015) and better health practices (Woods et al., 2012) exhibit fewer declines in inhibitory control and a slower decline in cognitive control.

Taken together, recent advances challenge the global decline approach by suggesting a domain-specific decline, affecting some cognitive-control processes more than others, with varying degrees of decline in different stages of ageing (Cabeza et al., 2018). These findings highlight the importance of considering specific domains when studying age-related changes and developing interventions to improve cognitive control in older adults.

That said, the lack of consensus on the definition of cognitive control contributes to the discussion about heterogeneity (Rey-Mermet and Gade, 2018; Heckner et al., 2021). For some researchers, cognitive control refers to the ability to focus attention and ignore distractions (Mayas et al., 2012), while for others it encompasses a wider range of functions. Some researchers use working memory (Zelazo et al., 2004) or attention to measure cognitive control, while others use inhibitory control (Persad et al., 2002; Salthouse et al., 2003; Vaughan and Giovanello, 2010). Theories need to account for these inconsistencies and form a more complex view of what cognitive control is and how its ageing should be measured.

### 3.2. Beyond the pre-frontal cortex

Despite strong evidence supporting the frontal lobe hypothesis of ageing, the theory does not explain all the neural effects and it is still a matter of scientific debate (Verhaeghen, 2011). To that end, researchers raised questions about whether declines in cognitive control result solely from frontal lobe changes and investigated the role of other relevant brain regions, such as the parietal and temporal lobes (Jimura and Braver, 2010; Vallesi et al., 2011). There are also knowledge gaps concerning the relation between declines in the frontal lobe and various environmental factors (e.g., lifestyle; Lövdén et al., 2010a).

Recent advances have attempted to challenge the focus on the frontal cortex. Large-scale neural changes in older adults’ regulation of default network (DN) activity have been related to the ageing of cognitive control (Park and Reuter-Lorenz, 2009; Grady, 2012). The DN refers to areas of the brain that are active when an individual is “at rest” or involved in tasks that are internal, such as self-reflective thoughts (Park and Reuter-Lorenz, 2009). When the brain moves to more demanding tasks, the DN is suppressed (Spreng and Turner, 2019). This suppression is reduced in older adults and this reduction is associated with underperformance on cognitive control tasks. Moreover, declines in DN activity and its functional connectivity have been associated with impaired resource allocation, accounting for observed age-related differences in cognitive performance (Park and Reuter-Lorenz, 2009).

In younger adult brains, neural networks are highly specialised, meaning they are densely connected within specific subnetworks but more sparsely connected between other networks (Chan et al., 2014; Geerligs et al., 2015). With age, networks become less specialised, as seen in a reduction of within-network connectivity and increase in internetwork activity both during specific tasks and in resting state conditions (Chan et al., 2014; Geerligs et al., 2015). The role of resting state networks and their functional segregation during cognitive control declines have been tested longitudinally. A recent study tested changes in brain networks over a 4-year period during older adulthood (Malagurski et al., 2020). The segregation of the DN, salience network, and frontoparietal control network deteriorated. Moreover, the drop in segregation of the frontoparietal control network was associated with declines in processing speed. Mixed patterns in network connectivity, including increases in DN and in between-network connectivity has been associated with strong cognitive functioning (La Corte et al., 2016). While resting state network activity has provided these insights and is useful for investigating whole-brain connectivity patterns, it has been recognised that an over-reliance on resting state data may withdraw from the focus on task-relevant cognitive measures, and the two should be used as complementary approaches (Campbell and Schacter, 2017).

Finally, older adults exhibit longer stop-signal reaction times compared to young adults and those age-related differences are associated with functional changes in the supramarginal gyrus, anterior insula, right inferior frontal cortex, and pre-supplementary motor areas (Coxon et al., 2016). These findings tie in with existing research showing the relationship between brain structure maintenance and improved cognitive control. For example, in task-switching studies, connectivity of task-relevant frontoparietal regions in younger adults was increased compared to older adults (Madden et al., 2010). Taken together, neural mechanisms underlying ageing of cognitive control goes beyond the functionality of the frontal cortex.

### 3.3. Plasticity and compensation

Another focal point in the neuroscience of cognitive-control ageing is plasticity—the ability of the neural networks to change and adapt in response to experiences and learning (Park and McDonough, 2013; Gutchess, 2014). Plasticity allows the brain to respond to environmental demands by way of structural changes that alter brain function and behaviour over time (Lövdén et al., 2010b; Lindenberger, 2018). Neuroplasticity models have helped to explain how structural changes occur due to a metaphoric mismatch between the scope of current functioning and the demands of the environment (Lövdén et al., 2010b; Lindenberger, 2018). This mismatch must occur for enough time and be intense enough to surpass a threshold, causing the changes necessary for a new state of balance between supply and demand (Lövdén et al., 2010b; Lindenberger, 2018; Lindenberger and Lövdén, 2019).

The exploration-selection-refinement (ESR) model of neuroplasticity posits three phases of learning (Lindenberger and Lövdén, 2019). Following heightened levels of activity, structural neural changes are supported by myelination and the formation of new dendric spines leading to an increase in new circuits formed and trialled. The best performing microcircuit is selected, variability decreases, and then the microcircuitry is stabilised and unselected microcircuits are withdrawn (Lindenberger and Lövdén, 2019).

Plastic changes in cognitive abilities may require both local plasticity (for example within the PFC) and widespread changes, such as improved network reorganisation aided by myelination of white matter tracts (Lindenberger and Lövdén, 2019). When considering neuroplasticity models in relation to neural activity patterns related to ageing, older age is more strongly associated with neural maintenance and cognitive flexibility than growth (Braver et al., 2014; Coxon et al., 2016; Kühn and Lindenberger, 2016), and neural patterns in older adults are less distinctive than in younger networks (Li et al., 2000). This could be due to an increased reliance on stronger network reorganisation which can be observed through increased bi-hemispheric activation (Cabeza, 2002).

Cabeza et al. (2002) theorised that if activity in specific regions of the brain declines with age, it would be reasonable to assume that compensatory mechanisms could gradually permit adaptive neurocognitive functioning to ageing of cognitive control. This idea has been supported by the notion of adaptations as driven by neurological supply deficits and environmental demand mismatches, which lead to plasticity-related changes in old age (Lövdén et al., 2010b). Increased neurological generalisation has been shown to counter neurocognitive declines, acting as a supportive adaptation (Cabeza et al., 2002; Park and McDonough, 2013).

Increasing age is associated with losses in controlled or fluid cognition (Zelazo et al., 2004; Thornton and Dumke, 2005; Wang et al., 2020). However, researchers have suggested older adults’ gains in crystalised knowledge (Kramer and Willis, 2002) could be due to shifts in cognitive architecture, with crystallised gains reflected in functional network architecture changes in the brain (Spreng and Turner, 2019). Increased reliance on prior knowledge has been highlighted as a compensatory mechanism that allows older adults to offset restrictions in cognitive resources, for example in decision making and planning tasks (Denburg and Hedgcock, 2015), and when strategically prioritising higher-value information in memory (Castel, 2007; Knowlton and Castel, 2022). Neural activity in older adults has shown increased recruitment of PFC regions and reduced suppression of the DN (Park and Reuter-Lorenz, 2009; Grady, 2012; Spreng and Turner, 2019). Thus, with age, cognition becomes increasingly influenced by existing knowledge, engaging the DN and lateral prefrontal brain regions to compensate for cognitive supply limitations (Lövdén et al., 2010b; Spreng and Turner, 2019).

Researchers have refined the compensatory model by examining how plasticity negates the effects of neural losses and promotes cognitive control in situations where cognitive demands are high (Vallesi et al., 2011; Park and McDonough, 2013; Cabeza et al., 2018). During demanding tasks older adults show increased bilateral activation in the frontal gyrus, the inferior parietal lobule, and posterior cerebellum. These activations are associated with over-recruitment of additional regions during more difficult tasks. Over-recruitment may lead older adults to reach their peak performance during difficult tasks at an earlier rate than younger adults, with quick improvements followed by plateaus in performance during inhibitory control tasks [for an explanation of neural activation and level of task demand, see the compensation-related utilisation of neural circuits hypothesis (CRUNCH), Reuter-Lorenz and Cappell, 2008; Vallesi et al., 2011; Reuter-Lorenz et al., 2021]. Thus, compensatory mechanisms include neural reorganisation in response to cognitive losses; upregulation of existing processes; and selection/promotion of an existing cognitive strategy without the development of a new structure or process (Cabeza et al., 2018).

## 4. Discussion

The reviewed research clarifies the need to provide a more nuanced understanding of the complex interaction between cognitive processes during ageing. To achieve this, we propose key areas of focus for future research, along with challenges in this field.

### 4.1. Neural plasticity

Studies on the role of plasticity and its relation to compensation during ageing suggest that the same brain regions can play different roles in older vs. younger adults depending on the connections with other intra-network regions. Yet, the structure of brain activity changes with age and their origins remain under debate and researchers have noted the need for any interpretations to be supported by behavioural evidence.

Building on the positive connections between behavioural measures, structural brain changes, and neural changes in functional connectivity (Lövdén et al., 2010a; Grady, 2012; de Lange et al., 2017), further examination of how neural plasticity underlies shifts in cognitive control during ageing holds promise for identifying ways to promote healthy ageing and maintain cognitive function throughout the lifespan. Specifically, similar to recent work in child development (Ossmy and Adolph, 2020), future ageing research would benefit from using advanced analytic techniques to (1) describe the heterogeneity in cognitive control ageing using a multivariate approach; (2) identify factors that contribute to different types of age-related declines; and (3) use identified factors and patterns to generalise findings from one group of older adults to another and help explain individual differences. When considering structural brain changes, researchers should also investigate structural invariance, as longitudinal stability has been associated with greater performance on different cognitive performance measures (de Frias et al., 2009).

### 4.2. Ageing cascades

Developmental researchers have increasingly examined child development as a cascadic effect, acknowledging that multiple processes and factors during development are at play during any given moment (Oakes and Rakison, 2019). This is also true for cognitive changes in later life. A person’s environment, behaviours, and cognitive resources continue to change from adulthood into old age, contributing to the significant individual differences in ageing.

The ageing research to-date has supported the notion of a multidimensional change in cognitive control with age and has acknowledged how individual experiences, such as education level, have been shown to impact brain network segregation (Chan et al., 2021). However, research has neglected a cascadic approach that considers how experiences influence ageing outcomes through a series of interconnected and reciprocal effects across multiple domains and their cumulative effect (Oakes and Rakison, 2019). Cognitive control is an ideal candidate for exploring ageing cascades because of the involvement of multiple processes and functions and their potential interactions (Oakes and Rakison, 2019). Specific attention should be paid to the cascading effects of life experiences on neural plasticity (Gutchess, 2014).

A longitudinal study design could be used to investigate the cascadic effects of life experiences on neural plasticity. As with developmental studies with infants and children, participants would be recruited from different age groups and researchers would follow them over an extended period, collecting data at multiple time points. Education, occupation, physical activity, social engagement, stress levels, and cognitive stimulation would be collected and analysed in a multivariate approach (i.e., full patterns from multiple data sources are used to predict changes in neural activity). Cognitive function could be assessed using neuropsychological tests, while neuroimaging techniques such as structural MRI, fMRI, and Diffusion Tensor Imaging would provide insight into brain structure and connectivity. Statistical analysis techniques, including regression models and mediation/moderation analyses, should be used to explore the relationships between life experiences and neural measures while accounting for confounding factors. Cross-sectional comparisons between individuals with diverse life trajectories and intervention studies, such as cognitive training programmes or physical exercise interventions, could further elucidate the impact of specific life experiences on neural plasticity in ageing.

### 4.3. Computational modelling

Despite much evidence from psychology and neuroscience, the exact mechanisms that contribute to age-related declines in cognitive control are still unclear. Computational modelling of cognition—the use of mathematical and computational approaches to simulate and explain neural and cognitive processes—has a high potential to achieve such understanding.

Previous work used computational modelling to formulate cognitive control as a process of controlling the flow of information between perceptual and executive systems, feeding internal system and external environment information to the controller (Haykin et al., 2012). In their review of the literature on computational modelling in ageing, Dully et al. (2018) discussed the useful nature of models for explaining individual differences by identifying age-associated processes related to multiple cognitive domains. One useful example is sequential sampling modelling, which explains decision making as a threshold that is passed once sufficient pre-determined sensory information is gathered, leading to a decision (Forstmann et al., 2016; Dully et al., 2018). In the context of cognitive-control ageing, sequential sampling models are useful in considering behavioural data associated with relevant latent variables such as the quantity and quality of evidence entering the decision-making process, and associated processes including sensory encoding and motor execution (Dully et al., 2018).

Utilising computational models could provide a quantitative framework for understanding the role of different brain regions and neurotransmitter systems in changes that occur with ageing and how they affect cognitive control performance. Moreover, simulating different ageing models and comparing the results to empirical data may provide the most likely explanations for observed changes in cognitive control with ageing, thereby guiding the development of interventions to maintain cognitive control in older adults. Finally, computational modelling facilitates communication and collaboration between researchers in different fields, such as cognitive neuroscience, psychology, and computer science, by providing a shared language and conceptual framework for understanding the ageing of cognitive control.

### 4.4. Whole brain connectivity patterns

Our final proposal for future direction involves the investigation of brain connectivity patterns at a whole-brain level. Recent advancements have initiated the exploration of how ageing affects whole-brain functional connectivity by examining patterns of segregation and integration throughout the entire brain (Park and Reuter-Lorenz, 2009; Campbell et al., 2013), as well as connectivity strength in relation to age and task performance (Lugtmeijer et al., 2023). This comprehensive approach holds significant promise for testing the role of age-related changes in brain connectivity in shaping cognitive control abilities (Geerligs et al., 2017).

By employing whole-brain analyses, researchers can unravel the intricate interactions and communication between segregated brain regions, shedding light on a spread of underlying mechanisms of cognitive control during ageing. Critically, it will enable the identification of specific networks or pathways that may be particularly susceptible or resilient to the effects of ageing. Furthermore, by considering the concept of cognitive reserve, which pertains to the brain’s capacity to cope with age-related decline through efficient neural processing or alternative strategies, researchers can gain insights into the individual differences observed in cognitive ageing trajectories. Finally, whole-brain approaches can potentially lead to the discovery of novel biomarkers and therapeutic targets for interventions aimed at promoting healthy cognitive ageing.

### 4.5. The challenges

Pushing the envelope of ageing research of cognitive control also comes with major challenges. One key challenge is the need for cross-sectional and longitudinal covariation studies. Such studies are crucial for capturing the dynamic nature of cognitive control and its changes across the lifespan. Examining both within-subject changes and individual differences in cognitive control abilities over time allows for identification of age-related trajectories and the exploration of potential predictors or moderators that contribute to individual differences in cognitive ageing.

Another critical challenge lies in unravelling the interplay between maturation, senescence, plasticity, and flexibility in cognitive control processes. The question of how age-related changes in brain maturation and senescence influence the contributions of plasticity and flexibility remains unanswered. Understanding these dynamics is crucial for developing effective interventions. Future research should aim to elucidate the complex interactions between these factors, providing insights into how cognitive control can be maintained or enhanced throughout the ageing process.

In conclusion, recent studies on the effects of typical ageing on cognitive control have revealed valuable insights into the complex interplay between brain function and cognitive abilities. However, there are still important knowledge gaps that concern the complexity of these effects in the space (whole brain networks) and in time (ageing cascades). Those knowledge gaps should be addressed by integrating computational, neural, behavioural perspectives, and methods from different disciplines to push the ageing cognition research forward.

## Author contributions

MD and OO contributed equally to conception and creation of this manuscript. Both authors contributed to manuscript writing, revision, read, and approved the submitted version.
